# Role of Hippo-YAP1/TAZ pathway and its crosstalk in cardiac biology

**DOI:** 10.7150/ijbs.47142

**Published:** 2020-07-06

**Authors:** Xiaoqing Chen, Wenchang Yuan, Yilang Li, Jiandong Luo, Ning Hou

**Affiliations:** 1 Key Laboratory of Molecular Target & Clinical Pharmacology, School of Pharmaceutical Sciences and the Fifth Affiliated Hospital, Guangzhou Medical University, Guangzhou 511436, China;; 2 KingMed School of Laboratory Medicine, Guangzhou Medical University, Guangzhou 511436, China.

**Keywords:** Hippo pathway, Wnt signaling, BMP signaling, GPCR signaling, cardiac biology

## Abstract

The Hippo pathway undertakes a pivotal role in organ size control and the process of physiology and pathology in tissue. Its downstream effectors YAP1 and TAZ receive upstream stimuli and exert transcription activity to produce biological output. Studies have demonstrated that the Hippo pathway contributes to maintenance of cardiac homeostasis and occurrence of cardiac disease. And these cardiac biological events are affected by crosstalk among Hippo-YAP1/TAZ, Wnt/β-catenin, Bone morphogenetic protein (BMP) and G-protein-coupled receptor (GPCR) signaling, which provides new insights into the Hippo pathway in heart. This review delineates the interaction among Hippo, Wnt, BMP and GPCR pathways and discusses the effects of these pathways in cardiac biology.

## Introduction

The Hippo pathway is a fundamental regulator that controls organ size, and first found in the *Drosophila* genus. Hippo, Salvador, Warts, Yorkie (Yki), and others were identified as the core components of the Hippo pathway also called Salvador-Warts-Hippo (SWH) pathway 1-3. The Hippo pathway is highly conserved, the homologs of which are found in mammals. This pathway mediates downstream genes expression to limit organ size and take part in tumorigenesis 4. Also, this pathway has been involved in cardiac biology including cardiogenesis, cardiac disease, and cardiac regeneration, which is mediated by cardiomyocyte proliferation, differentiation, apoptosis and others 5. Hearts with the inactivation of Hippo pathway are subject to abnormal development, while the regeneration activity of heart and cardiomyocytes is impaired by YAP1/TAZ deficiency 6, 7. Recent studies have revealed that multiple crosstalk among Hippo-YAP1/ TAZ, Wnt/β-catenin, Bone morphogenetic protein (BMP) and G-protein-coupled receptor (GPCR) signaling, which enriches the regulation mechanism of the Hippo pathway and the role of Hippo pathway in other signaling. In this review, we briefly recapitulate important new discoveries related to possible interactions among Hippo, Wnt, BMP and GPCR pathways, highlighting the crosstalk of these pathways in cardiac biology.

## Hippo-YAP1/TAZ Pathway and Its Role in Cardiac Biology

**Figure 1:**
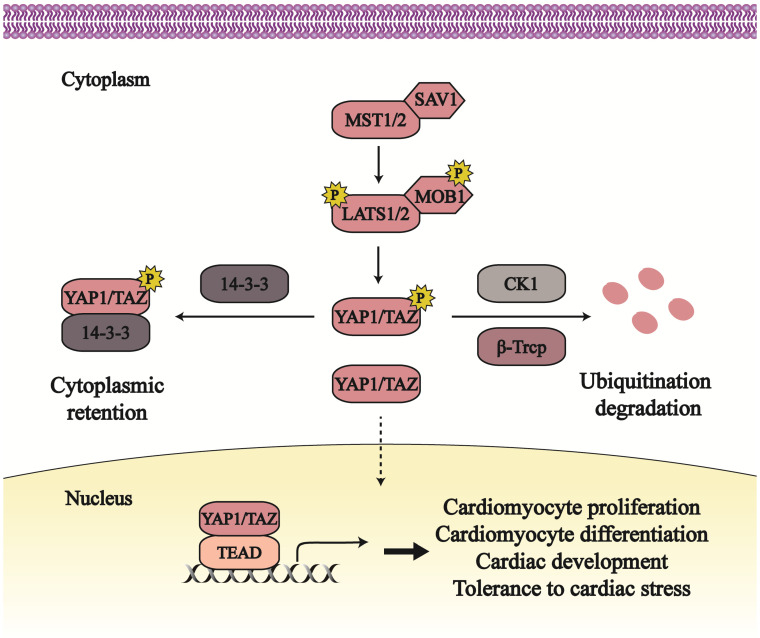
** The overview of Hippo**-**YAP1/TAZ pathway and its role in cardiac biology.** The Hippo-YAP1/TAZ pathway mainly consists of MST1/2, SAV1, LATS1/2, MOB1 and two downstream effectors. The Hippo pathway, as conserved pathway in organ-size control, plays pivotal role in cardiomyocyte growth, cardiac development, cardiac disease and so on. Line arrows indicate activation, whereas dotted lines mean translocation.

In mammal, mammalian sterile 20-like protein kinases 1/2 (MST1/2) are the homologs of Hippo in *Drosophila*, phosphorylated and activated by upstream stress. Active MST1/2, together with its partner Salvador homolog 1 (SAV1), phosphorylates and activates large tumor suppressor kinase 1/2 (LATS1/2; homologs of Warts) and Mps one binder kinase activator-like 1A/1B (MOB 1A/1B; ortholog of Mats) 8, 9. The active LATS1/2, binding to its adaptor MOB, facilitates the phosphorylation and suppression of two downstream effectors, Yes-associated protein 1 (YAP1; ortholog of Yorkie) and its paralog transcriptional coactivator with PDZ-binding motif (TAZ) (Figure 1).

LATS1/2 can phosphorylate YAP1 at Ser61, Ser109, Ser127, Ser164, and Ser381, and target at Ser66, Ser89, Ser117, and Ser311 of TAZ 10. While YAP1 Ser127 (Ser89 in TAZ) is phosphorylated, YAP1/TAZ binds to 14-3-3 proteins that contribute to YAP1/TAZ export from the nucleus and its retention in the cytoplasm 11. YAP1 Ser381 phosphorylation (Ser311 in TAZ) triggers further phosphorylation by Casein kinase 1 isoform δ/ε (CK1 δ/ε), and consequently results in ubiquitination degradation dependent on the β-transducin repeat-containing E3 ubiquitin protein ligase complex (SCF^β-TRCP^) 12. YAP1/TAZ phosphorylation by the Hippo pathway has an inhibitory effect on its activity. And this inhibition can be released through Nemo-like kinase (NLK)-induced phosphorylation at YAP1 Ser128 13.

When the Hippo pathway is in an off state, YAP1/TAZ translocates to the nucleus, where they combine with transcriptional enhanced associate domain proteins (TEADs) to facilitate gene transcription and generate downstream output. Other transcriptional partners also were found to interact with YAP1/TAZ to regulate transcription, such as Smad, and p63/p73 14-16. The activation of YAP1/ TAZ mainly mediates cell proliferation and apoptosis, further regulating cancer growth and organ size 4, 17.

As a highly conversed pathway, the Hippo pathway plays an essential role in controlling heart size and development 18, 19 (Figure 1). A lack of the Hippo pathway in embryonic hearts causes cardiac hypoplasia and lethality 7, 20. SAV1 deficiency releases YAP1 from phosphorylation and then increases cardiomyocyte (CM) proliferation, which thickens ventricular walls, enlarges ventricular chambers, and leads to ventricular septal defect 7. Mouse hearts without LATS2 or MST1/2 are characterized with the similar phenotypes. Embryos with YAP1 knockdown are hard to survive past embryonic day 16.5, subjecting to aberrant cardiac growth 21. Except for heart growth, the Hippo signaling in adult hearts also contributes to resistance of cardiac stresses and improvement of heart survival. In response to cardiac stresses such as myocardial infraction and pressure overload, MST1 and LATS1/2 both are increased and activated to enhance CM apoptosis and reduce autophagy, subsequently leading to cardiac systolic and diastolic dysfunction 20, 22, 23. Increasing the active form of YAP1 in the neonatal heart can rescue left ventricular tissue from myocardial infraction and improve myocardial tissue 24.

The Hippo pathway mainly mediates cell proliferation and differentiation to affect cardiac biology; YAP1/TAZ, as the downstream effectors of the Hippo pathway, facilitates the transcription of relative target genes, such as *Pik3cb, Dhrs3, Sox17* and so on. Lacking of Pik3cb impedes embryonic survival; the phosphoinositide 3 kinases-protein kinase B (PI3K-AKT) signaling is activated and promotes CM proliferation via YAP1-induced Pik3cb expression 25, 26. The differentiation activity of subepicardial cells is impaired due to enhanced Dhrs3, an inhibitor of the retinoic acid pathway 27, 28. This may lead to the lethality of LATS1/2 cardiac knockout embryos with active YAP1 and enhanced Dhrs3 27. Also, YAP1 suppresses another differentiation-relative gene *Sox17* that mediates the differentiation action of human embryonic stem cells 29. CM proliferation and differentiation dependent on the Hippo pathway contribute to normal cardiac size and structure; and then YAP1/TAZ endows CMs with a primitive and fetal condition that increases CM tolerance to stresses and is beneficial to CM and heart survival.

## Interaction of Hippo-YAP1/TAZ Signaling with the Wnt Pathway

The Wnt proteins can activate Wnt/β-catenin signaling (also called canonical Wnt signaling), Wnt/Ca^2+^ signaling, and other Wnt-related pathways. β-catenin, as an intracellular signal transducer, is pivotal in canonical Wnt signaling. In the off state, β-catenin is trapped in a destruction complex composed of AXIN, adenomatous polyposis coli (APC), Casein kinase 1 α (CK1α), and glycogen synthase kinase 3β (GSK 3β), where β-catenin is phosphorylated by CK1α and GSK 3β in turn 30. Subsequently, the phosphorylated β-catenin suffers from ubiquitination and degradation by SCF^β-TRCP^. Wnt binds to Frizzled (FZD) and Lipoprotein receptor-related protein 5/6 (LRP5/6) to initiate the Wnt signaling, consequently disassembling destruction complex 31. β-catenin escapes from the β-catenin destruction complex into the cytoplasm and undergoes nuclear translocation to initiate target gene transcription, while other components of destruction complex are recruited to the plasma membrane. β-catenin initiates target gene transcription mostly alongside with T-cell factor/lymphoid enhancing factor (TCF/LEF) transcriptional partner 30.

**Figure 2:**
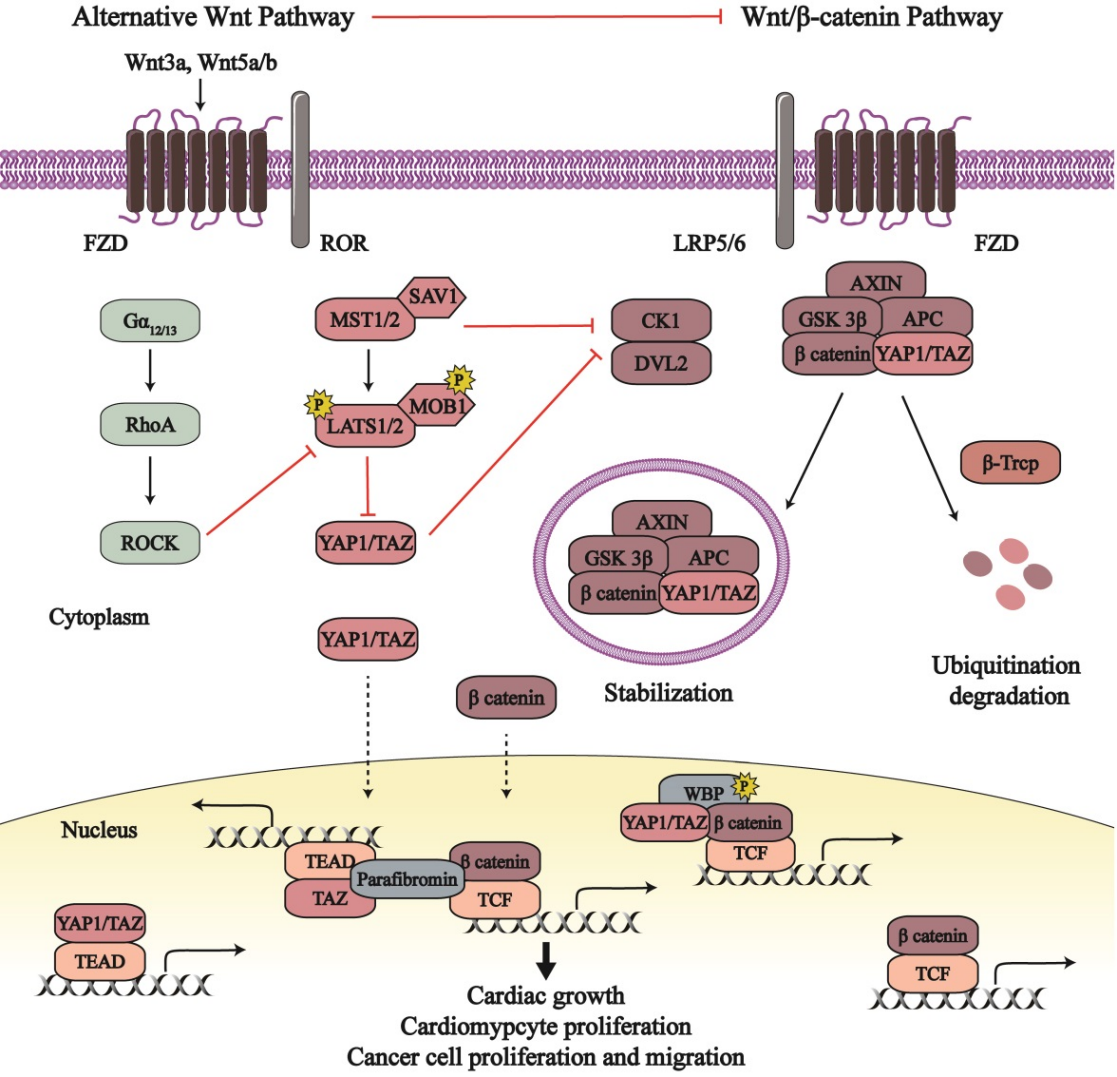
** The crosstalk between the Hippo**-**YAP1/TAZ pathway and Wnt signaling.** The Wnt/ β-catenin signaling and alternative Wnt signaling participate in the modulation of the Hippo-YAP1/TAZ pathway. Conversely, the Hippo-YAP1/TAZ pathway plays a role in the regulation of Wnt signaling. The crosstalk between the Hippo pathway and Wnt signaling affects cancer biological behavior, cardiomyocyte proliferation and cardiac growth. Line arrows indicate activation, whereas connector lines imply inhibition; dotted lines mean translocation.

Numerous studies have revealed crosstalk between the Hippo pathway and Wnt signaling (Figure 2). In canonical Wnt/β-catenin signaling, CK1ε (another member of the CK1 family) combines with and activates dishevelled 2 (DVL2) to transduce biological signals from the Wnt receptors. They, as positive regulators, destabilize β-catenin degradation complex and recruit the complex to cell membrane in order to reduce β-catenin degradation and promote its activity 32, 33. MST1/2, a core serine/threonine kinase in the Hippo pathway, inhibits canonical Wnt/β-catenin signaling independent on YAP1/TAZ 34. MST1/2 binds to CK1ε, subsequently disrupting the relationship between DVL2 and CK1ε as well as impeding the CK1ε-induced phosphorylation and activation of DVL2 34. This MST1/2-CK1ε interaction suppresses cell proliferation, which can be recovered by Wnt3a treatment. Expect for MST1/2, YAP1/TAZ also can disturb CK1ε-DVL2 binding. Phosphorylated TAZ combines with DVL2 and therefore inhibits the interaction of DVL2 and CK1ε; TAZ deficiency in mouse enhances nuclear β-catenin and target gene transcription 35. SAV1, another member of Hippo pathway, was found to inactive β-catenin through the Hippo pathway. β-catenin activation in the heart of SAV1-deficient embryos enhances cell proliferation, expansion of ventricular myocardial layers, and thickening of ventricular walls, whereas β-catenin deficiency reduces SAV1 CKO-induced cardiac expansion 7. Therefore, active Hippo-YAP1/TAZ pathway dampens Wnt/β-catenin signaling and its proliferation effects in cardiac growth.

In basal epidermal cells, YAP1-5SA, an active form of YAP1, overexpression enhances active β-catenin in the nucleus, leading to enhanced cell proliferation 36. As mentioned in section 2, YAP1 can upregulate Pik3bc and then activate PI3K-AKT pathway in CM 25. YAP1-S112A (constitutively active YAP1) inhibits GSK 3β activity in the embryonic heart by activating the insulin-like growth factor-PI3K/AKT pathway; subsequently, reduced phosphorylation of β-catenin by GSK 3β leads to enhanced β-catenin activity 25, 37. β-catenin undertakes positive mediators in CM proliferation 38. As result, YAP1 may indirectly mediate Wnt/β-catenin signaling via enhancing the activity of PI3K/AKT pathway, which can affect CM proliferation. Moreover, YAP1-induced activation of canonical Wnt/β-catenin signaling may rescue AC16 human CMs after ischemia-reperfusion (IR) injury 39. So, YAP1-dependent β-catenin activation results in the promotion of CM proliferation and survival, contributing to cardiac growth. And YAP1 and β-catenin synergistically increase CM proliferation by respective transcriptional target genes, which mediate cardiac development and heart diseases.

Recent reports have found that YAP1/TAZ is combined into the β-catenin degradation complex, which affects Wnt/β-catenin pathway and the Hippo pathway at the same time. In the absence of Wnt, YAP1/TAZ binds to the β-catenin degradation complex and the latter recruit SCF^β-TRCP^ for the degradation and devitalization of YAP1/TAZ and β-catenin 40, 41. Following Wnt ligand stimulation, both YAP1/TAZ and β-catenin escape from the disassemble degradation complex, subsequently translocate to the nucleus, and interact with different transcription factors (such as TEADs and TCF/LEF) to produce respective downstream effects. What's more, the YAP1/TAZ-β-catenin destruction complex can be stranded in the multi-vesicular body compartment to protect themselves against degradation, as so to exert their biological output 42. Except for combining into the β-catenin degradation complex, cytoplasmic YAP1/TAZ directly binds to β-catenin and retains the latter in the cytoplasm to inhibit β-catenin nuclear accumulation 43. This direct binding is conducive to balance between Hippo pathway and Wnt/β-catenin signaling in neurogenesis and tumorigenesis 43, 44. In the nucleus, YAP1/TAZ and β-catenin bind to not only their respective transcription factors but also each other to stimulate transcriptional actions. With the Wnt pathway in the on state, the YAP1/TAZ-β-catenin complex combines with the phosphorylated WW domain-binding protein 2 (WBP2), enters the nucleus, and provokes transcriptional action by the β-catenin-TCF complex 45. Besides, the TAZ-β-catenin complex associates with parafibromin (a nuclear scaffold protein) which cooperatively initiates TEAD and TCF co-activator activities 46.

Interestingly, a growing body of evidence indicates that Wnt proteins can activate YAP1/TAZ, independent of Wnt/β-catenin signaling in many cell lines 47-51. An alternative Wnt pathway has been promulgated, consisting of Wnt, FZD/ROR, Ga_12/13_, Rho GTPases, LATS1/2, and YAP1/TAZ, wherein YAP1 and TAZ are the key downstream transcription cofactors 49. The Wnt5a/b and/or Wnt3a, as extracellular stimulants, activate FZD-receptor tyrosine kinase-like orphan receptor 1/2 (ROR1/2) receptor complex to transmit biological signals. Therewith, this stimulates the inhibitory effect of the Ga_12/13_-Rho GTPases axis in LATS1/2, which increases the nuclear sequestration and activity of YAP1/TAZ. Furthermore, *Wnt5a/b* are identified as target genes of YAP1/TAZ, which suggests the existence of a positive feedback loop in alternative Wnt-YAP1/TAZ signaling to reinforce alternative Wnt pathway 49.

This fascinating axis regulates several biological effects, including osteogenic differentiation, cell migration, cell polarity, and cytoskeletal oscillation. Wnt3a activates the Wnt-YAP1/TAZ pathway to maintain the pluripotency of bovine trophoblast stem cells (TSCs) 50. The Wnt5a-induced YAP1/TAZ activation mediates cell polarity and formation of organ primordia 47. Wnt-activated YAP1 regulates rostral and caudal brain tissue identity and affects neural tissue from human-induced pluripotent stem cells (hiPSCs) 51. However, there are no more studies about the relationship between Wnt-YAP1/TAZ pathway and cardiac biology.

When the alternative Wnt-YAP1/TAZ pathway is activated, there is increased expression of Wnt/β-catenin signaling inhibitors including Wnt5a, Dickkopf-related protein 1 (DKK1), BMP4, connective tissue growth factor (CTGF), and cysteine-rich angiogenic inducer 61 (CYR61), all of which attenuate canonical Wnt/β-catenin signaling 49, 52. However, the afore-mentioned studies describe that active YAP1/TAZ can boost β-catenin activity in canonical Wnt signaling, and directly participate in β-catenin transcriptional events as a co-transcriptional factor. This controversy implies more detailed crosstalk between the Hippo-YAP1/TAZ pathway and Wnt/β-catenin signaling that needs further investigation to understand this sophisticated regulatory system.

## Interaction of Hippo-YAP1/TAZ Signaling with the BMP Pathway

**Figure 3:**
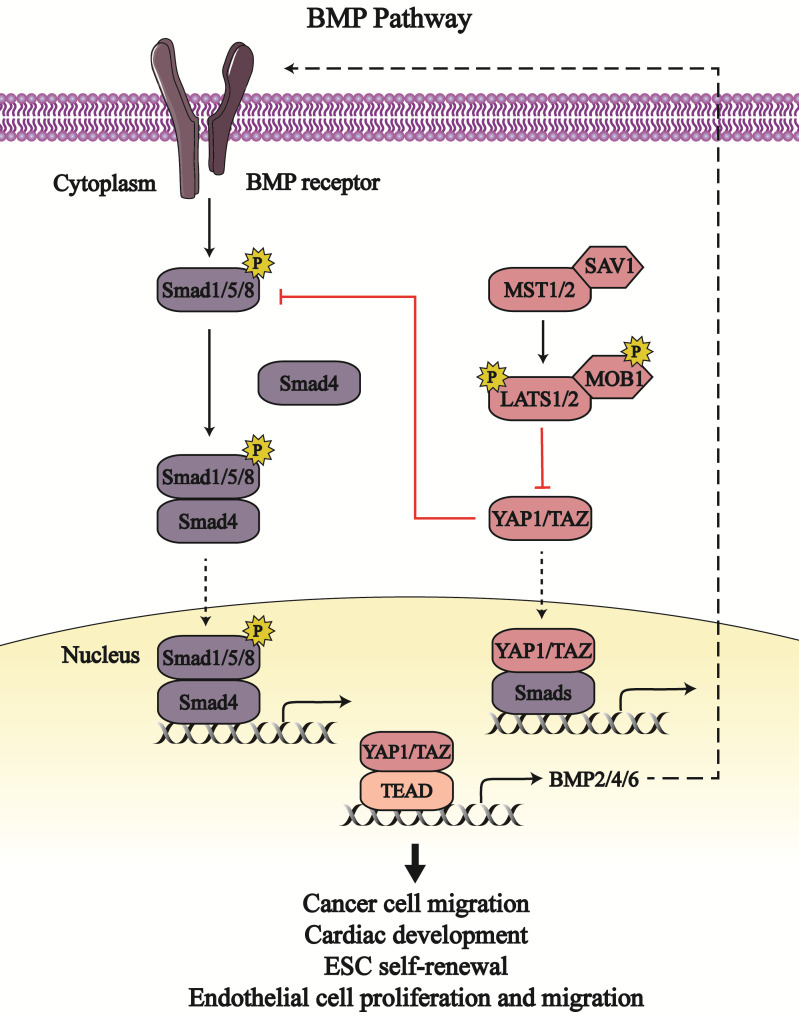
** The crosstalk between the Hippo**-**YAP1/TAZ pathway and BMP signaling.** The Hippo-YAP1/TAZ pathway bidirectionally regulate the bone morphogenetic proteins (BMP) pathway. YAP1/TAZ binding to Smads form transcription complex to regulate respective output in cancer cells, embryonic stem cells (ESC), endothelial cells as well as heart. Line arrows indicate activation, whereas connector lines imply inhibition; dotted lines mean translocation.

Bone morphogenetic proteins (BMPs) belong to the family of transforming growth factor-β (TGF-β) and are involved in embryonic development as well as organ and tissue homeostasis. BMPs associate with type 1 and 2 cell membrane receptors to activate and phosphorylate intercellular receptor-regulated Smads (Smad1, Smad5, and Smad8) that combine with common partner Smads (Smad4) and accumulate in the nucleus 53. The Smads complex binds with transcriptional factors to regulate target gene expression 54. The BMP pathway and Hippo signaling pathway intersect to regulate embryonic growth and maintain cell homeostasis (Figure 3).

In *Drosophila*, the transcription activity of the Smads complex is potentiated due to impaired Yki activity, which elicits defective escort-cell germline differentiation 55. In human and mouse mesenchymal stromal/stem cells (MSCs), active YAP1 inhibits the phosphorylation of Smad1, Smad5, and Smad8, and downregulates the expression of BMP signaling target genes, such as *inhibitor of DNA-binding proteins* (*Id1*, *Id2*, and *Id3*) 56. The BMP-induced Ids output can regulate cell differentiation; definitively, Id1 can restrain the differentiation and sustain self-renewal activity of embryonic stem cells (ESC) 57. So, YAP1 may downregulate Ids expression via BMP signaling as so to affect embryonic self-renewal capacity. Moreover, YAP1/TAZ inhibits BMP signaling through the augmented expression of BMP antagonists, such as CTGF, cysteine-rich motor neuron 1 protein (CRIM1), Follistatin, BMP and activin membrane-bound inhibitor homolog (BAMBI), E3 ubiquitin-protein ligase SMURF1/2, and Noggin 58, 59. Thus, YAP1/TAZ enhances the proliferation, migration and barrier function of endothelial cells to prevent bleeding in response to mechanical stretch by upregulating BMP antagonists as well as downregulating BMP signaling 58.

Several BMPs have been identified as target genes of YAP1/TAZ, including BMP2, BMP4 and BMP6. YAP1/TAZ can enhance *BMP2* transcription and expression that are inhibited by MOB1/2 and LATS1/2 60, 61. Enhanced BMP2 impairs the function and homeostasis of intestinal epithelial cells 61. Moreover, BMP2 induces epithelial-to-mesenchymal transition (EMT) in the cardiac cushion, which is conducive to the development of valve and septa 54. YAP1-mediated BMP2 expression results in the generation of the venous pole contributing to the growth of heart atrium 60. BMP4 is upregulated by YAP1 transcription coactivator in zebrafish and mouse endothelial cells 62, 63. Following BMP4 stimulation, active BMP signaling induces the apoptosis of human umbilical vein endothelial cells and dampens endothelial tubulogenesis 64. However, BMP4 treatment enhances the proliferation and migration of mouse embryonic endothelial cells and human microvascular endothelial cells via activation of vascular endothelial growth factor/vascular endothelial growth factor receptor 2 (VEGF/VEGFR2) signaling 65. These differences of BMP signaling in endothelial cells may be caused by the different downstream signaling of BMP4 and cell lines used. Also, YAP1-dependent BMP6 expression contributes to endothelial cell migration and tube formation via activating BMP pathway 66. As such, the Hippo pathway stimulates BMP signaling by increasing the content of BMPs, which maintains endothelial cell functions and cardiac development.

Hereditary hemorrhagic telangiectasia (HHT) is a vascular syndrome characterized by vascular malformations 67. Among the five subtypes of HHT, HHT5 is caused by *GDF2* (encoding BMP9) mutations 68. In response to BMP9 stimulation, YAP1 translocates to the nucleus to form a complex with Smad1/5, and facilitates *CTGF/CYR61* transcription in endothelial cells; this mechanism partly explains the etiology of HHT 69. Except for Smad1/5, YAP1/TAZ can bind to Smad2/3 in the nucleus to enhance Smad2/3 nuclear accumulation, which improves BMP signaling activity and EMTs 69, 70. Notably, YAP1 and BMP are both activated in the neural crest of avian embryos; active YAP1 is indispensable for BMP activity, whereas BMP is sufficient for YAP1 activity in neural crest migration 71. To the extent, there exists a positive and bidirectional interaction between BMP pathway and YAP1/TAZ.

## Interaction of Hippo-YAP1/TAZ Signaling with the GPCR Pathway

**Figure 4:**
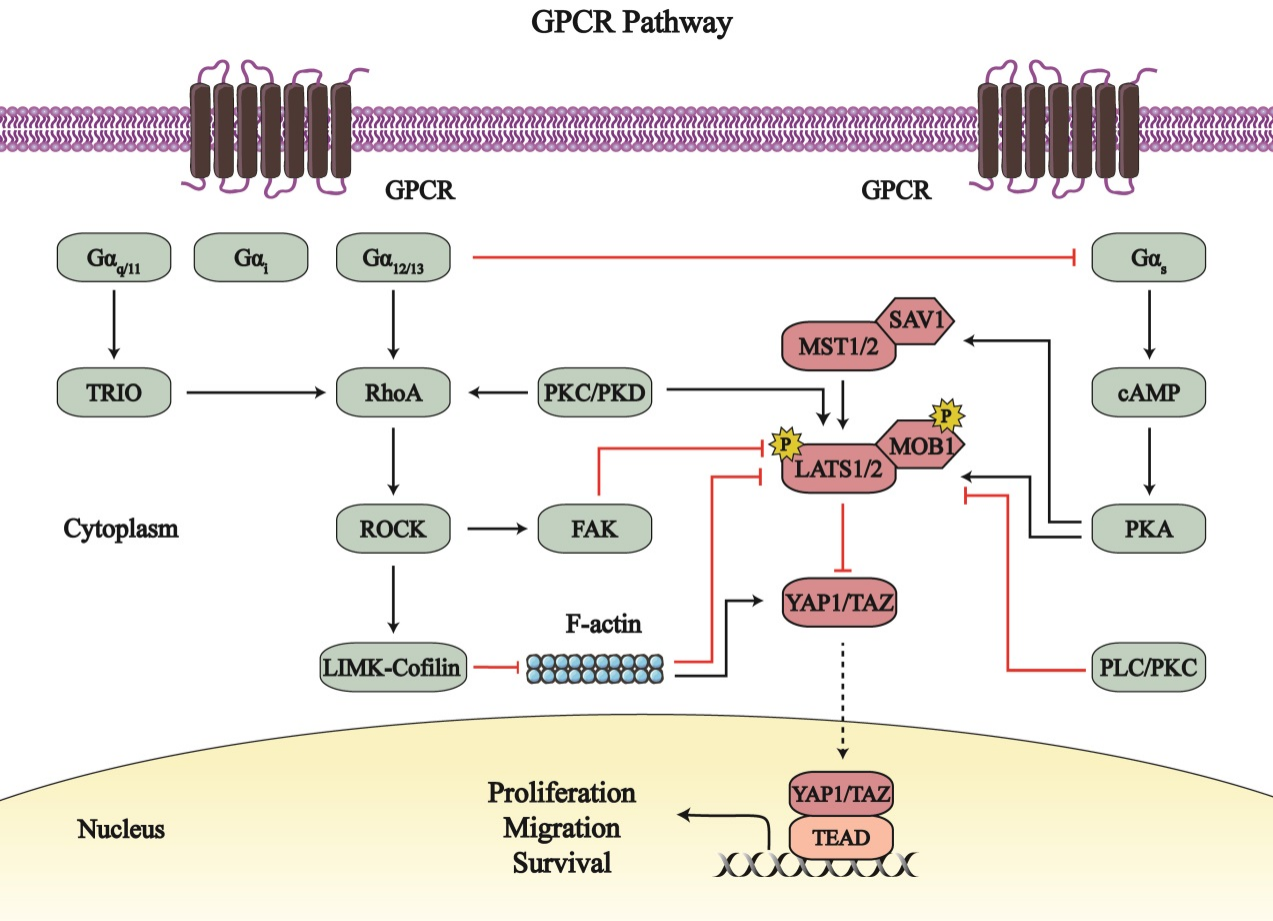
** The crosstalk between the Hippo**-**YAP1/TAZ pathway and GPCR signaling.** G-protein-coupled receptors (GPCR) signaling regulate the Hippo-YAP1/TAZ pathway by serval G proteins and different downstream signaling. Gα_12/13_, Gα_q/11_, and Gα_i_, together with RhoA-ROCK axis and PKs axis, mainly upregulate YAP1/TAZ; while Gα_s_ generally inhibits YAP1/TAZ by PKA axis. GPCR signaling-dependent Hippo pathway regulation affects the proliferation, migration and survival of cancer cells, endothelial cells and cardiomyocytes. Line arrows indicate activation, whereas connector lines imply inhibition; dotted lines mean translocation.

G-protein-coupled receptors (GPCRs) are a type of cell-surface transmembrane receptors that contact intercellular G proteins to regulate second messengers to elicit downstream effects 72, wherein the Hippo-YAP1/TAZ signaling pathway has garnered attention (Figure 4).

The Hippo-YAP1/TAZ pathway is regulated by different heterotrimeric G proteins. YAP1/TAZ is generally activated by Gα_12/13_, Gα_q/11_, and Gα_i_; these G proteins are coupled with different GPCRs, including lysophosphatidic acid receptors, sphingosine1-phosphate (S1P) receptors, and protease-activated receptor 1 73-79. However, Gα_s_ inhibits the Hippo-YAP1/TAZ pathway by other GPCRs, such as free fatty acid receptor 1/4 (FFAR1/4, also called as G-protein receptor 40/120 [GPR40/120]) 80-82.

Gα_12/13_, Gα_q/11_, and Gα_i_ mainly upregulate Ras homology family member A (RhoA)-Rho associated protein kinase (ROCK) signaling to catalyze the phosphorylation of its substrates, which affects YAP1/TAZ. The LATS1/2-induced phosphorylation of YAP1/TAZ is suppressed by the Gα_12/13_-RhoA-ROCK pathway, with the net effect of increased YAP1/TAZ nuclear location and activity 73, 74. Activated YAP1 potentiates the transcription and expression of the glycolytic enzyme phosphoglycerate mutase (PGAM1) as well as c-Myc in osteosarcoma cells, which may contribute to the reprogramming of glucose metabolism in the tumor 74. In addition to canonical Hippo signaling, the Gα_12/13_-RhoA-ROCK axis regulates F-actin and myosin assembly to activate YAP1/TAZ 78, 79, 83. Specifically, the G-protein-coupled estrogen receptor 1 (GPER; also known as GPR30) acts via the Rho-ROCK signaling to mediate the LIM-domain kinase (LIMK)-Cofilin pathway that stabilizes F-actin and promotes cytoskeleton assembly; both F-actin and myosin activation activates YAP1/TAZ, independent on LATS1/2 inhibition 83. YAP1/TAZ activation by the Gα_12/13_-RhoA-ROCK axis can promote endothelial cell sprouting, facilitate vascular smooth muscle cell (VSMC) migration and proliferation, and may participate in embryonic vascular development 73. Furthermore, Rho-ROCK axis promote trophectoderm differentiation and inhibit the formation of the inner cell mass through enhanced YAP1 nuclear localization 84. Thus, the Gα_12/13_-RhoA-ROCK axis may participate in YAP1/TAZ-mediated cardiac biology.

Likewise, Gα_q/11_, and Gα_i_ mediate RhoA-ROCK signaling to affect the Hippo pathway. The Gα_q_ activates TRIO-RhoA asix (non-canonical Gα_q_-signaling) in uveal melanoma. The Gα_q_-TRIO-RhoA axis promotes focal adhesion kinase (FAK)-regulated MOB1 Tyr26 phosphorylation, which disrupts the inhibition of the MOB1-LATS complex on YAP1; simultaneously, this axis enhances YAP1 Tyr357 phosphorylation to activate YAP1 and facilitate cell growth 75. The S1P-S1P receptor (S1PR) pathway acts via Gα_i_ to stabilize YAP1 and promote the survival of pancreatic progenitor cells 85. However, the results about S1PR-induced YAP1 activation are different from reports from Kemppainen Lab. The S1PR2-Gα_12/13_ axis stimulated YAP1 and enhanced its transcriptional activity in response to 1 h treatment with sphingosylphosphorylcholine (SPC); however, longer SPC treatment (9 h) led to higher levels of phosphorylated YAP1 Ser127, and lower levels of its target genes 86. With short SPC treatment, S1PR2 may activate Gα_q_, Gα_12/13_, and Gα_i_ subunits to dephosphorylate and stabilize YAP1 85, 86. During long SPC treatment, S1PR2 may indirectly affect the Gα_s_ downstream pathway via Gα_12/13_, so that LATS1/2 is activated and phosphorylated YAP1 Ser127 is increased 86, 87. And there exists a negative feedback mechanism that YAP1/TAZ increase endogenous LATS2 activity through direct induction of TEAD-dependent *Lats2* transcription 88. These special mechanisms may lead to the long SPC treatment-induced suppression of YAP1.

Except for RhoA- ROCK axis, other signaling also mediates YAP1/TAZ. In triple-negative breast cancer (TNBC) cells, bisphenol S impacts the GPER-activated phospholipase C/protein kinase C (PLC/PKC) pathway, which decreases the levels of phosphorylated-LATS1/2 and subsequently increases YAP1 dephosphorylation 89. Therefore, PLC/PKC-mediated YAP1 activation promotes TNBC cells migration. P2Y_2_ receptor (P2Y_2_R) is a purinergic GPCR that regulates Gα_q/11_-PKC signaling to control the flow of Ca^2+^ and K^+^ ions 90. The P2Y_2_R-PKC axis activates YAP1 by inhibiting LATS1/2 and subsequently promote cell proliferation in human c-Kit^+^ cardiac progenitor cells (hCPCs), which helps to rescue CM from damages 91, 92. Moreover, the GPCR agonist neurotensin attenuates YAP1 phosphorylation at Ser127/397 and augments YAP1 nuclear localization via PKD signaling 93. Most importantly, PK-induced YAP1/TAZ activation is divided into two phases 94. In the first phase, the GPCR-PKC/PKD axis induces rapid activation of LATS1/2, to enhance the phosphorylation of YAP1 at Ser127. Next, the GPCR-PKC/PKD axis activates RhoA and induces the formation of the actin cytoskeleton, and subsequently dephosphorylates YAP1 and promotes YAP1-TEAD activity.

Paradoxically, PKs, together with Gα_s_, impairs the activity of YAP1/TAZ. In combination with Gα_s_, FFAR1/2/4 activates PKA-induced phosphorylation of MST1/LATS1; thereafter, YAP1 is phosphorylated and detained in the cytoplasm, which inhibits cell proliferation and cell metastasis, and induces apoptosis of tumor cells 80-82. Notably, the Gα_s_-PKA-induced suppression of YAP1 can be disrupted by positive G proteins, including Gα_12/13_, Gα_q/11_, and Gα_i_ 95. GPCRs exert bidirectional influence on the Hippo-YAP1/TAZ pathway, which is mutually restricted. However, the relationship between GPCRs and the Hippo-YAP1/TAZ pathway in cardiac biology is unclear.

## Conclusion

Accumulating reports focus on the Hippo-YAP1/TAZ signaling in cardiac biology. As an important heart-control signaling, the Hippo pathway dysregulation contributes to abnormal cardiac development and cardiac diseases. In this review, we discuss the crosstalk between Hippo pathway and other signaling including Wnt/β-catenin, BMP and GPCR signaling. Wnt signaling and GPCR signaling mainly act as the upstream regulation of the Hippo-YAP1/TAZ pathway, affecting the activity of its core members as well as directly mediating YAP1/TAZ subcellular location and abundance. The Hippo signaling pathway also controls the output of BMP signaling and Wnt/β-catenin signaling by regulating their kinases activity and transcriptional events. Such, the interaction among these pathways may result in the role of the Hippo pathway in cardiac biology, which may explain the complex output of Hippo pathway in heart and cardiomyocytes. Accordingly, the crosstalk between Hippo pathway and other signaling sheds light on physiological events of cardiac development and pathophysiological mechanism of cardiac diseases.
